# Childhood trauma as a transdiagnostic risk factor: clinical implications and preventive interventions

**DOI:** 10.1192/j.eurpsy.2024.81

**Published:** 2024-08-27

**Authors:** R. J. Bou Khalil

**Affiliations:** Psychiatry, Saint-Joseph University, Beirut, Lebanon

## Abstract

**Abstract:**

Abstract: This presentation seeks to explore the interplay between various types of psychological traumas and their potential correlation with the development of distinct types and severities of eating disorders. Emphasis will be placed on elucidating the underlying biological underpinnings and psychological and developmental factors that contribute to the manifestation of diverse eating disorder phenotypes in individuals who have experienced childhood maltreatment.

Drawing upon existing research and novel insights, I will present some data from studies investigating the notion that the observed variations in eating disorder presentations may be linked especially to environmental influences. Contrary to the conventional focus on genetic determinants, our findings suggest that the differential ecophenotypic expression of eating disorders may not solely be attributed to DNA variants but rather to the complex interplay between genetic predispositions and environmental contexts.

In particular, I will expose the concept of an ecophenotype characteristic of eating disorders associated with childhood maltreatment, positing that the unique ecological context in which an individual is raised significantly influences the trajectory and severity of their eating disorder. This exploration extends beyond a mere examination of genetic markers, shedding light on the environmental and ecosystemic factors that shape the development of an individual’s relationship with food and body image.

**Disclosure of Interest:**

None Declared

